# *Chlamydomonas* Axonemal Dynein Assembly Locus *ODA8* Encodes a Conserved Flagellar Protein Needed for Cytoplasmic Maturation of Outer Dynein Arm Complexes

**DOI:** 10.1002/cm.21206

**Published:** 2015-02-07

**Authors:** Paurav B Desai, Judy R Freshour, David R Mitchell

**Affiliations:** Department of Cell and Developmental Biology, SUNY Upstate Medical UniversitySyracuse, New York

**Keywords:** flagella, motility, cilia, dynein, assembly, intraflagellar transport

## Abstract

The *Chlamydomonas reinhardtii oda8* mutation blocks assembly of flagellar outer dynein arms (ODAs), and interacts genetically with *ODA5* and *ODA10*, which encode axonemal proteins thought to aid dynein binding onto axonemal docking sites. We positionally cloned *ODA8* and identified the gene product as the algal homolog of vertebrate LRRC56. Its flagellar localization depends on ODA5 and ODA10, consistent with genetic interaction studies, but phylogenomics suggests that LRRC56 homologs play a role in intraflagellar transport (IFT)-dependent assembly of outer row dynein arms, not axonemal docking. ODA8 distribution between cytoplasm and flagella is similar to that of IFT proteins and about half of flagellar ODA8 is in the soluble matrix fraction. Dynein extracted *in vitro* from wild type axonemes will rebind efficiently to *oda8* mutant axonemes, without re-binding of ODA8, further supporting a role in dynein assembly or transport, not axonemal binding. Assays comparing preassembled ODA complexes from the cytoplasm of wild type and mutant strains show that dynein in *oda8* mutant cytoplasm has not properly preassembled and cannot bind normally onto *oda* axonemes. We conclude that ODA8 plays an important role in formation and transport of mature dynein complexes during flagellar assembly. © 2014 The Authors. Cytoskeleton Published by Wiley Periodicals, Inc.

## Introduction

Cilia serve important functions as motile organelles in most eukaryotic organisms, and also serve as sensory signaling platforms even when motility has been lost, as seen in many cell types of multicellular metazoa, including vertebrates. This broad range of functions has stimulated much recent work on mechanisms that regulate the trafficking of proteins to and from the ciliary compartment [Hsiao et al., 2012]. Here we focus on the assembly pathway of axonemal outer dynein arms (ODAs), because they are the most abundant structural components of motile ciliary axonemes (next to tubulin itself), rely on intraflagellar transport (IFT) for their assembly [Hou et al., 2007; Ahmed et al., 2008] and thus serve as an easily assayed cargo for revealing essential properties of this trafficking machinery. In addition, loss of ciliary motility in the majority of human primary ciliary dyskinesia (PCD) cases has been linked to defects in axonemal dynein ATPase assembly [Lobo et al., 2014]. The genetic defects underlying these assembly errors occur in genes that encode dynein subunits themselves [Pennarun et al., 1999; Olbrich et al., 2002; Loges et al., 2008], axonemal docking site proteins [Panizzi et al., 2012; Jerber et al., 2013; Hjeij et al., 2014], or proteins involved in cytoplasmic pre-assembly and transport of this ca. 2 mDa dynein motor [Omran et al., 2008; Duquesnoy et al., 2009; Loges et al., 2009; Horani et al., 2012; Kott et al., 2012; Mitchison et al., 2012; Hjeij et al., 2013; Horani et al., 2013; Moore et al., 2013; Tarkar et al., 2013; Zariwala et al., 2013; Onoufriadis et al., 2014]. Thus the identification of conserved proteins important for ODA assembly provides new candidate genes for human PCD.

Most dynein assembly proteins were first identified by taking advantage of genetic analysis in the haploid unicellular alga *Chlamydomonas reinhardtii*, in which we and others have characterized nearly twenty loci needed for ODA assembly (*oda* loci). Most *Chlamydomonas oda* loci have been cloned, and their molecular identification and functional characterization have formed the basis for understanding the roles of their vertebrate homologs in the overall pathway of axonemal dynein assembly. The *Chlamydomonas* ODA complex, as typical of ciliary ODA motors, consists of over 15 subunits, including three catalytic heavy chains, two intermediate chains, and at least 10 light chains. Although many *oda* loci encode one of these subunits, several encode assembly factors and have been useful in understanding the pathway of axonemal dynein assembly [Koutoulis et al., 1997; Takada et al., 2002; Casey et al., 2003; Wirschell et al., 2004; Freshour et al., 2007; Omran et al., 2008; Mitchison et al., 2012; Dean and Mitchell, 2013]. We have now determined the molecular identity of one assembly locus that had not been previously cloned, *oda8*.

Over 25 years ago [Kamiya, 1988], analysis of genetic interactions among the many loci needed for assembly of ODAs in *Chlamydomonas* divided all loci identified at that time into three groups, on the basis of their non-complementation in temporary zygotic diploids (dikaryon analysis; see [Dutcher, 2014]). One group of such interacting loci is now known to encode subunits of the doublet-associated ODA docking complex (ODA-DC) [Fowkes and Mitchell, 1998], and a second group includes loci that encode either dynein arm subunits [Fowkes and Mitchell, 1998] or cytoplasmic proteins needed for dynein arm subunit stability and pre-assembly in the cytoplasm [Omran et al., 2008; Duquesnoy et al., 2009; Mitchison et al., 2012]. The third group includes three loci, *oda5, oda8*, and *oda10*. Kamiya [1988] reported poor complementation in dikaryons formed between *oda5* and *oda10* gametes and between *oda8* and *oda10* gametes, and somewhat reduced complementation between *oda5* and *oda8* gametes, whereas all three of these mutants fully complement with any of the other *oda* mutations analyzed. *ODA5* [Wirschell et al., 2004] and *ODA10* [Dean and Mitchell, 2013] both encode axonemal coiled-coil proteins with weak similarity to *Chlamydomonas* DC subunits. The ODA5 protein was hypothesized to interact with ODA10 and form an accessory DC for doublet attachment of ODAs, and ODA8 was hypothesized to interact with this accessory complex [Wirschell et al., 2004], but we have recently determined that ODA10, unlike the ODA-DC, is not essential for dynein attachment to axonemal binding sites [Dean and Mitchell, 2013]. ODA5 and ODA10 assemble independently of the ODA-DC in flagella [Wirschell et al., 2004; Dean and Mitchell, 2013] and the ODA-DC also assembles normally in the flagella of *oda5, oda8* or *oda10* mutant strains [Takada and Kamiya, 1994; Wakabayashi et al., 2001; Wirschell et al., 2004; Owa et al., 2014], so the failure of dynein assembly in these three mutants is clearly not due to lack of the ODA-DC. Here we report on the molecular identity of the *ODA8* locus, examine the basis of the genetic interaction of *oda8* with *oda5* and *oda10*, and clarify the stage of dynein assembly affected by these mutations. ODA8 has properties consistent with a role in the maturation and transport of ODA complexes during flagellar assembly, and depends on ODA5 and ODA10 for this function. We conclude that it belongs in a new category of assembly factors, those needed for cytoplasmic maturation of axonemal outer arm complexes into assembly-competent cargos for IFT-dependent transport, and into binding-competent complexes for attachment to doublet microtubules.

## Results

### ODA8 is an LRR Protein With a Conserved Dynein Assembly Function

**Figure 1:**
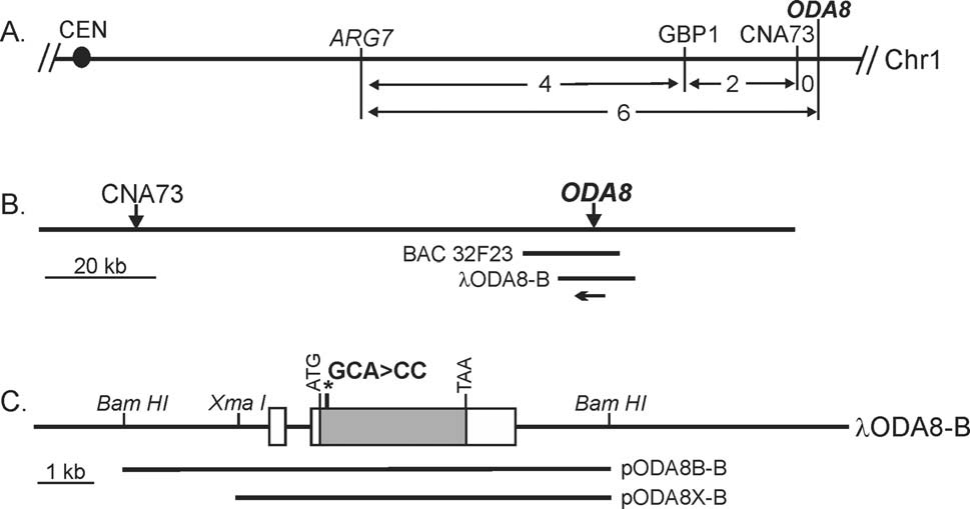
Molecular identification of the *oda8* locus. (A) To positionally clone *ODA8*, based on its map location on the right arm of chromosome 1, the segregation of markers GBP1 and CNA73 was followed in six meiotic products with crossovers in the *ARG7-ODA8* interval. Numbers indicate the number of crossover events observed in each sub-interval. (B) Location of BAC and lambda phage clones that rescued the slow swimming phenotype of an *oda8* mutant. Arrow indicates the orientation of the *ODA8* gene. (C) The location of cloned subfragments of the ODA8-B phage insert that rescued the *oda8* phenotype are shown below a diagram of the *ODA8* gene structure, which also shows the frame-shift mutation identified at codons 35–36 of the *oda8-1* allele.

The *Chlamydomonas ODA8* locus is defined by three allelic mutations that disrupt assembly of flagellar ODAs and map to the right arm of chromosome 1 [Kamiya, 1988]. To identify the *ODA8* gene, we tested molecular markers for proximity to *oda8* by following marker segregation in products from a genetic cross between an *arg7, oda8* strain and a polymorphic wild type strain. In six products with crossovers between *arg7* and *oda8*, crossovers were also observed between *oda8* and markers PBT302, GBP1 and RB47, but no crossovers were seen between *oda8* and CNA73 (Fig. 1A). Based on our previous analysis of the relationship between genetic distance and physical distance in this region [Freshour et al., 2007], sequences within 200 kb of CNA73 were considered as candidates for *ODA8*, and genes in this interval were examined for characteristics of flagellar genes. Only one gene, located 100 kb telomeric to CNA73, was identified as a likely candidate based on the appearance of homologs only in organisms with motile cilia. This gene was designated as *MOT37* (Protein ID: 146778) by the *Chlamydomonas* genome project [Merchant et al., 2007]. Transformation of a 17.5 kb BAC clone (PTQ12187) that spans *MOT37* into an *oda8* strain fully rescued the motility and dynein assembly defects back to wild type, as did introduction of plasmids containing only the *MOT37* gene (Fig. 1B). As further confirmation that the *oda8* phenotype resulted from a mutation in *MOT37*, we sequenced fragments of the *MOT37* gene that were amplified from mutant DNA, and identified a frame-shift mutation in codon 35 (Fig. 1C) that results in a premature translational stop. Since less than 5% of the full length protein would be produced by this truncated coding region, the *oda8-1* allele should be considered a null mutation.

Because previously deposited database sequences for the *MOT37* mRNA (XM_001690129) were incomplete, we verified gene structure through direct sequence analysis of our genomic clones, RT-PCR of mRNA isolated from deflagellated cells, and comparison of our genomic sequences with RNASeq data. Our analyses confirm the most recent sequence assembly (v5.0) and gene structure predictions for this gene, reported at Phytozome (http://www.phytozome.net) as Cre01.g043650.t1, and show that the gene encodes a 921 residue leucine-rich repeat (LRR) protein homologous to vertebrate LRRC56. No functional studies of LRRC56 members have been previously reported. Based on current nomenclature for dynein-related proteins in *Chlamydomonas* with LRRs, this gene is now formally designated *DLU2* [Hom et al., 2011]. For continuity, and to retain an association with its role in outer arm dynein assembly, we will refer to the locus as the *ODA8* locus and the encoded protein as ODA8. Two other LRR proteins have known roles in outer row dynein function, LC1 and ODA7. LC1 is a dynein light chain associated with an ODA heavy chain, HCγ [Benashski et al., 1999], and is known to be essential for ODA assembly in trypanosomes [Baron et al., 2007] and ciliates [Kutomi et al., 2012], whereas ODA7 is a cytoplasmic factor essential for correct cytoplasmic pre-assembly of outer arm dynein [Duquesnoy et al., 2009; Mitchison et al., 2012]. However, the LRRC56 family does not appear to be closely related at the primary sequence level to either of these other LRR families.

**Figure 2:**
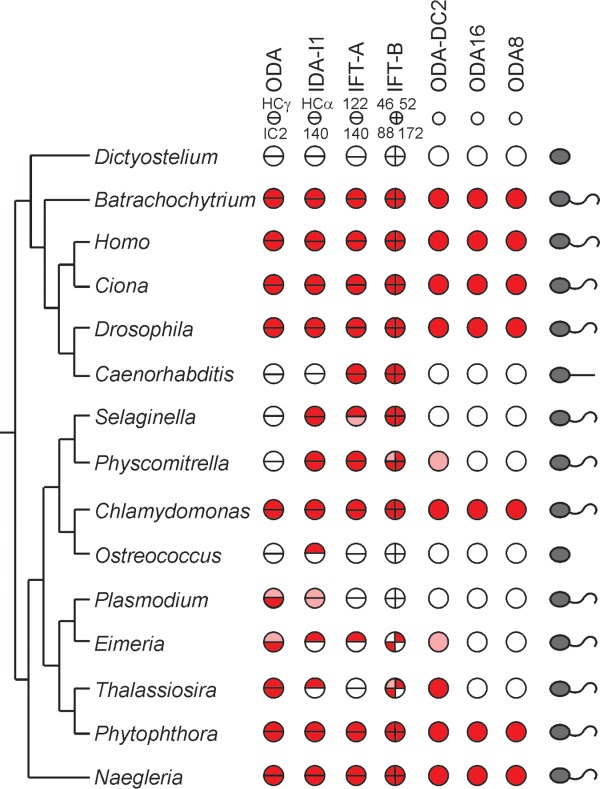
Phylogenomic distribution of ODA8/LRRC56 gene orthologs. The presence of orthologs of genes important for ciliary assembly (IFT-A and IFT-B proteins), ODA assembly (ODA-DC2, ODA16 and ODA8) and axonemal dynein subunits (ODA, IDA-I1) is diagrammed in selected organisms spanning diverse eukaryotic clades. Specific proteins used in these comparisons as representative of each multi-subunit complex are listed in a key across the top, with each sector of a circle representing a single protein. Orthologs were identified as sequences generating reciprocal BLAST best hits, starting with the *Chlamydomonas* protein sequence; stronger similarity is represented by dark shading, weaker but significant similarity by light shading, and failure to identify any ortholog is shown as unshaded segments. The presence or absence of cilia and the motility state of cilia, if present, is diagrammed along the right margin. Actual E-values for each comparison are available in Supporting Information Table I.

Phylogenomic analyses can reveal potential roles of otherwise uncharacterized genes by showing retention or loss that coincides with that of other genes or characteristics [Briggs et al., 2004]. Such a plot for *LRRC56/ODA8* genes (Fig. 2 and Supporting Information Table I) shows that homologs are lost from organisms that lack motile cilia and retained in organisms with motile cilia, as expected, but with two notable exceptions. First, no ODA8 homologs are found in organisms that show secondary loss of ODAs, such as the mosses *Selaginella moellendorffii* and *Physcomitrella patens*, consistent with a dedicated role in ODA assembly or function. Second, ODA8 homologs are also missing from organisms that retain outer arms but that do not require IFT for ciliary assembly, such as the diatom *Thalassiosira pseudonanna* or the apicomplexans *Plasmodium falciparum* and *Eimeria tenella*. A similar pattern is seen for genes encoding IFT-associated outer arm dynein assembly adaptor WDR69/ODA16, but not for a docking complex subunit (DC2). We therefore sought to characterize the phenotype of *oda8* mutant cells and the biochemical properties of the ODA8 protein to see if they supported a hypothesized role for this gene product in IFT-dependent dynein assembly or alternatively, if the ODA8 protein functioned as previously hypothesized by interacting with ODA5 and ODA10 as part of an accessory DC on the surface of doublet microtubules [Kamiya, 1988, Fowkes and Mitchell, 1998, Wirschell et al., 2004].

### ODA8 Has a Unique Biochemical Distribution Dependent on ODA5 and ODA10

**Figure 3:**
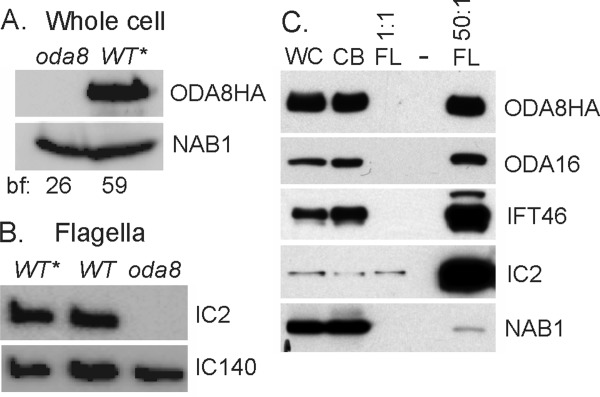
ODA8 distribution is similar to that of transport factors. (A) HA-tagged ODA8 expressed from an integrated transgene rescues the beat frequency (bf) of *oda8* cells to wild type levels (∼60 Hz), and the expressed protein migrates at ∼104 kDa on blots of transformant oda8::ODA8HA (WT*) whole cell protein samples. (B) Expression of the HA-tagged transgene rescues the assembly of flagellar ODA subunit IC2 to wild type levels. Anti-IC140 recognizes inner arm I1 dynein, which is not affected by the *oda8* mutation and serves as a loading control. C. Samples of whole cells (WC), deflagellated CB and flagella (FL) from equal numbers of cells (1:1) and with flagella at 50-fold excess (50:1) were probed with the indicated antibodies. ODA8HA abundance ratios are similar to those of IFT-B subunit IFT46 and IFT-associated transport factor ODA16, not those of ODA subunits such as IC2. Anti-NAB1, which recognizes a 26 kDa cytoplasmic protein, was used as a loading control in A and C.

To see if ODA8 is a flagellar protein important for dynein docking, we expressed a modified version of the gene in which the last three codons were replaced by sequences encoding three copies of the HA epitope tag. This construct can rescue *oda8* mutant strains to wild type swimming velocity (150 µm/s for wild type (sd = 38, n = 37), 58 µm/s for *oda8* (sd = 18, n = 27), and 148 µm/s (sd = 30, n = 19) for *oda8* rescued with the tagged gene), normal flagellar beat frequency and wild type ODA assembly levels (Figs. 3A and 3B). When such tagged cells (hereafter WT* cells) were deflagellated and the relative abundance of ODA8 in flagella vs. deflagellated cell bodies (CB) was examined, about 50-fold more protein was found in cytoplasmic fractions than in flagella (Fig. 3C). This distribution is similar to that of IFT-associated assembly factor ODA16 and IFT-B subunit IFT46, but unlike that of axonemal ODA intermediate chain subunit IC2 or cytoplasmic protein NAB1. Based on this distribution, ODA8 is likely to have a specific function in both cytoplasmic and flagellar compartments.

**Figure 4:**
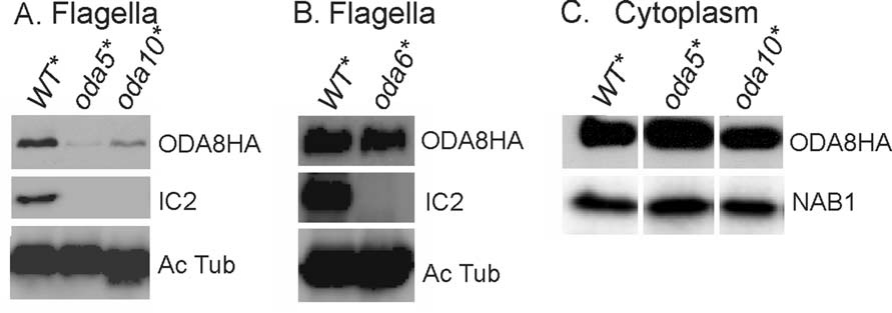
Interacting mutants alter ODA8 distribution. (A) Blots of flagella from WT* and from doublet mutant *oda5, oda8* and *oda10, oda8* strains that express ODA8HA (oda5* and oda10*) were probed with antibodies to HA and IC2. Both *oda5* and *oda10* block ODA assembly, and both greatly reduce the flagellar abundance of ODA8HA. B. Blots of flagella from WT* and from a double mutant *oda6,oda8* strain expressing ODA8HA (oda6*) show that flagellar abundance of ODA8HA is not affected by the failure of ODA assembly in *oda6*. C. Blots of cell extracts show that cytoplasmic abundance of ODA8HA is unaffected by the *oda5* and *oda10* mutations. Acetylated tubulin (AcTub) is used as a loading control for flagellar samples, and NAB1 for cell extract samples. All three lanes in both blots in C. were from the same blot images. Vertical spaces indicate removal of intervening lanes.

To test the prediction that ODA8 is dependent on ODA5 and ODA10 for its flagellar localization, we constructed strains that carry *oda5* or *oda10* mutations, as well as the *oda8* mutation, and that express the ODA8HA transgene (designated oda5* and oda10*). Flagellar levels of ODA8HA were reduced to less than 10% of wild type levels in either the *oda5* or *oda10* background (Fig. 4A), supporting the model that ODA8 depends on ODA5 and ODA10 for its transport into the flagellar compartment and/or its association with doublet microtubules. Flagella of *oda6, oda8* cells expressing ODA8HA (oda6*), which lack ODAs due to the *oda6* mutation (a null mutation in the IC2 gene) do not show a reduction in the amount of ODA8HA (Fig. 4B) confirming that the reduced level of ODA8HA in oda5* and oda10* flagella is specifically due to the *oda5* and *oda10* mutations and not due to absence of outer arm dynein. To see if the reduced levels of ODA8HA in oda5* and oda10* flagella could be due to instability of ODA8 in the absence of ODA5 or ODA10, we blotted cytoplasmic extracts of the oda5* and oda10* strains and probed for HA. As shown in Fig. 4C, there was no change in the amount of ODA8HA in the cytoplasm of either oda5* or oda10* mutant strains, indicating that ODA8 protein stability is not dependent on the *ODA5* or *ODA10* gene products. Instead, the ODA5 and ODA10 proteins must play a role in the transport and/or anchoring of ODA8 in flagella.

**Figure 5:**
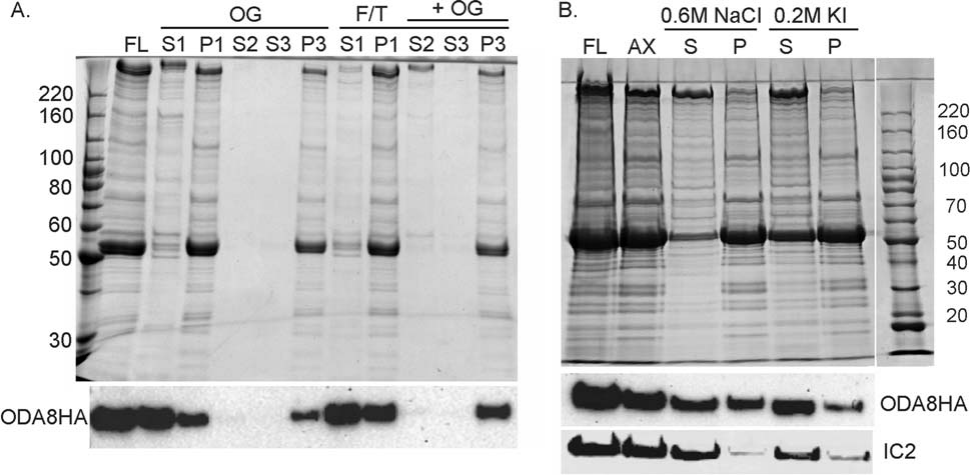
Flagellar ODA8 is in both matrix and axonemal fractions. (A) Coomassie stained gel (upper panel) and blot probed for HA (lower panel) of WT* FL, supernatant fractions (S) and pellets (P) after three sequential extractions of flagella with solutions containing 0.8% octylglucopyranoside detergent (OG), by treating flagella to a freeze/thaw (F/T), or to freeze/thaw followed by 0.8% OG (+ OG). About half of the flagellar ODA8HA is solubilized by detergent or freeze/thaw treatment. (B) Coomassie stained gel and blots of whole FL, 0.1% NP-40 detergent-extracted axonemes (AX) and the supernatant and pellet fractions of axonemes extracted with the indicated salt concentrations. About half of axonemal ODA8HA resists extraction with 0.6 M NaCl, unlike ODA subunit IC2. The sizes in kDa of protein standards are shown along the gel margins.

We next fractionated flagella isolated from WT* cells to determine whether flagellar ODA8 was present in the matrix fraction, as expected of an IFT-associated protein [Cole et al., 1998] or axonemal, as expected for a protein interacting with ODA5 and ODA10 [Wirschell et al., 2004; Dean and Mitchell, 2013]. When flagellar membranes were removed by treatment with 0.8% non-ionic octylglucoside detergent (OG), over half of ODA8HA became soluble but a substantial fraction remained with the axonemal pellet (Fig. 5A, OG lanes). When this pellet was treated serially with two additional OG treatments, no additional ODA8HA appeared in supernatant fractions. Thus ODA8HA is present in both detergent soluble (membrane + matrix) and insoluble (axonemal) pools of flagellar proteins. To test if ODA8HA in the membrane + matrix fractions is associated with the membrane, matrix or both, flagella from WT* cells were treated to freeze/thaw cycles, which physically disrupt membrane integrity and release matrix proteins without solubilizing most of the membrane proteins. A similar proportion of ODA8HA was released when flagella from WT* cells were fractionated by detergent or by freeze–thaw (Fig. 5A, F/T lanes). Subsequent treatment of the F/T pellet with detergent (Fig. 5A, F/T+OG lanes) failed to release additional ODA8HA. Thus flagellar ODA8HA is associated with both matrix and axonemal pools of flagellar protein, but not with the flagellar membrane.

Salt extraction was used to determine whether interaction of ODA8 with axonemes could be directly dependent on a physical interaction with its genetically interacting partners, ODA5 and ODA10. Both ODAs and the ODA5 and ODA10 proteins are quantitatively extracted by treatment of axonemes with 0.6M NaCl [Wirschell et al., 2004, Dean and Mitchell, 2013]. When flagella from WT* cells were demembranated with 0.1% NP-40 and the resulting axonemes were extracted with 0.6M NaCl, most of the outer arm dynein was solubilized, as expected, whereas nearly half of the axonemal ODA8HA could still be pelleted (Fig. 5B). Therefore, once assembled onto axonemes, ODA8 apparently no longer depends on ODA5 and ODA10 for its doublet microtubule association. Most of this axonemal pool of ODA8HA was solubilized by treating axonemes with 0.2M KI.

### ODA8 is Not Needed for *In Vitro* Binding of Axonemal ODA Complexes Onto Axonemal Docking Sites

**Figure 6:**
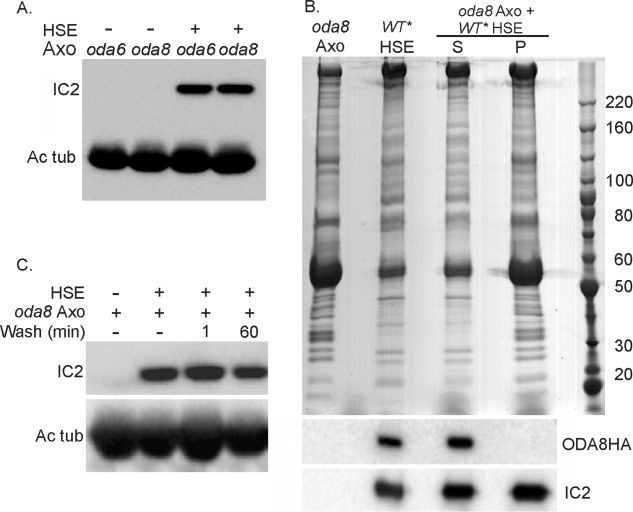
ODA8 is not needed for ODA binding to axonemes *in vitro*. (A) Axonemes from the indicated strains were mixed with a dialyzed salt extract from wild type axonemes (HSE). After 1 h, axonemes were pelleted and the presence of bound dynein was tested by blotting for IC2. (B) Coomassie stained gel and corresponding blots of *oda8* axonemes (Axo), a salt extract of WT* axonemes (HSE), and the supernatant (S) and pellet (P) fractions prepared 1 h after mixing *oda8* axonemes and HSE at a 1:2 ratio. IC2 pellets with the *oda8* axonemes, whereas ODA8HA remains soluble. (C) The stability of ODAs bound to *oda8* axonemes (as in A) was tested by resuspending the pellet fraction for the indicated times before re-pelleting. IC2 remains tightly bound after a 60 min wash. Acetylated tubulin (Ac tub) blots show equal loading of axonemal protein (A and C).

To see if ODA8 is necessary for dynein binding to axonemes *in vitro*, a wild type axonemal salt extract was mixed with *oda6* axonemes, which should retain the axonemal fraction of ODA8 (Fig. 4B), and *oda8* axonemes, which lack ODA8. As illustrated in Fig. 6A, ODA complexes can bind equally well to either axoneme sample. As a further test of the role of ODA8 in dynein binding, we prepared a salt extract from WT* axonemes, in which the ODA8 protein is epitope tagged, and mixed the desalted extract in 2-fold excess with *oda8* mutant axonemes. As shown in Fig. 6B, the ODA8HA that is extracted with NaCl failed to bind to *oda8* mutant axonemes, whereas ODAs (IC2) pelleted with the *oda8* axonemes at an approximately normal stoichiometry. To test the affinity of these re-bound outer arms for their binding sites on *oda8* axonemes, the pellet fraction from a binding assay was either processed directly as a gel sample (as in Figs. 6A and 6B), washed briefly in HMDEK or resuspended in HMDEK for 1 h before a second spin. Washing, irrespective of duration, did not alter the amount of dynein bound (Fig. 6C). We conclude that dynein from a wild type flagellar extract can rebind to *oda8* axonemes in a stable manner without the continued presence of ODA8, and thus the ODA8 protein is not essential for high affinity dynein binding to axonemes under these *in vitro* conditions, even though it is required for normal assembly of dynein onto axonemes *in vivo*.

### Pre-Assembled Cytoplasmic ODA Complexes Are Defective in *oda8* Mutant Cells

**Figure 7:**
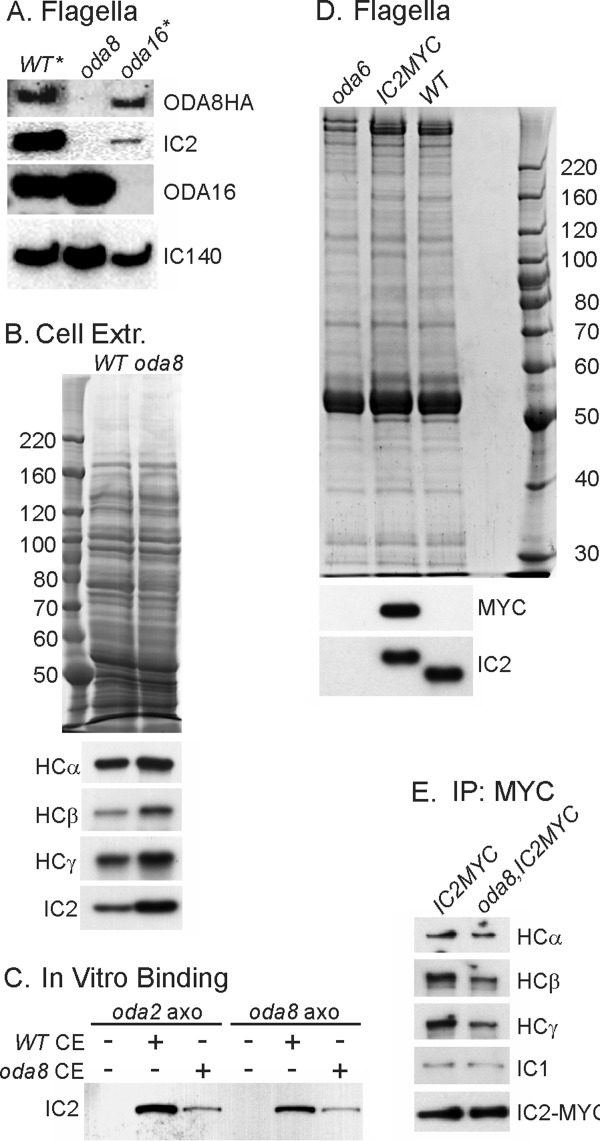
ODA complexes are defective in *oda8* cytoplasm. (A) Blots of flagella from WT*, *oda8*, and double mutant strain *oda8, oda16* that expresses the *ODA8HA* transgene (oda16*) probed with the indicated antibodies. Neither mutation prevents flagellar assembly of the other protein. IC140 is used as a loading control. (B) Stained gel and corresponding blot of cell extracts from wild type and *oda8* cells, loaded for equal amounts of protein. Blots show the relative abundance of ODA subunits in each extract. (C) Binding experiment in which extract samples shown in B, adjusted to contain equal ODA subunit concentrations, were mixed with axonemes from the indicated *oda* mutants. After 1 h, axonemes were pelleted, washed, and prepared as gel samples. An immunoblot shows the relative amount of IC2 bound. (D) Gel and corresponding blots of flagella from *oda6* (IC2-null)*, oda6* transformed with a MYC-tagged IC2 gene (*IC2MYC*), and untransformed wild type cells (*WT*), probed for MYC and IC2, show that MYC-tagged IC2 supports normal levels of ODA assembly. (E) Blots of anti-MYC IP samples, from cell extracts of *IC2MYC* and *oda8, IC2MYC*, probed with the indicated antibodies. Both IC1 and IC2-MYC are present at equal abundance, whereas the relative amount of each heavy chain is reduced in the *oda8* sample to about 55% of its wild type abundance.

Although we previously observed no genetic interaction between *oda8* and *oda16* by dikaryon rescue analysis [Ahmed and Mitchell, 2005], the phylogenomic co-distribution of ODA8 and ODA16 (Fig. 2), and fractionation of much flagellar ODA8 with matrix (Fig. 5), suggested that ODA8 and ODA16 might work at the same stage of IFT-dependent ODA transport. If so, we considered it possible that lack of one protein might alter transport to the flagellar compartment of the other, or maintenance of its normal flagellar abundance. Direct blot analysis of the effects of *oda8* and *oda16* mutations on flagellar abundance of ODA16 and ODA8 proteins (Fig. 7A) confirms that neither protein depends on the other for its transport into the flagellar compartment or for maintaining its normal flagellar abundance.

One alternative explanation for the binding results with axonemal extracts(Fig. 6) would be that dynein is modified in an ODA8-dependent manner during its initial *in vivo* assembly, but that ODA complexes, once successfully assembled, can be salt extracted in a form that no longer requires ODA8 for axonemal re-binding. If such a modification occurred during transport to flagellar assembly sites, or at the time of final assembly onto doublet microtubules, ODA complexes in the cytoplasm might have axonemal binding properties that were not yet affected by lack of ODA8. In wild type cytoplasm, ODAs have pre-assembled into complexes that contain all five major ODA subunits [Fowkes and Mitchell, 1998], and these complexes can bind to *oda2* mutant axonemes (which lack ODAs due to an outer arm dynein heavy chain defect) *in vitro* [Ahmed et al., 2008]. As shown in Fig. 7B, dynein from *oda8* mutant cytoplasm contains normal or slightly elevated levels of the major outer arm dynein subunits, consistent with our previous analyses [Fowkes and Mitchell, 1998]. To see if these proteins are assembled into a form that can bind to outer doublet docking sites, extract samples containing equal amounts of ODA subunits were incubated, at a 2:1 stoichiometric ratio, with axonemes from *oda8* or from ODA heavy chain mutant *oda2*. After 1 h on ice, axonemes were pelleted, resuspended briefly in buffer, re-pelleted, and prepared as gel samples. Immunoblots of these samples showed much less IC2 bound from *oda8* cell extracts than from wild type cell extracts (Fig. 7C). Multiple experiments produced a range of values for the relative amount of IC2 bound from *oda8* extracts that varied from 8% to 25% of the wild type levels, based on quantification of signals by either densitometry of films or direct measurement of chemiluminescent signals using a digital detector. In contrast, little difference was seen between binding to *oda2* vs. *oda8* axonemes, regardless of the source of extract. These results suggested that ODA8 might play a role during the maturation of dynein in the cytoplasm into an assembly-competent complex, and prompted a re-examination of the assembly state of ODA proteins in *oda8* cytoplasm.

We reported previously that at least five ODA subunits (three heavy chains and two intermediate chains) have pre-assembled in the *oda8* cytoplasm, based on their co-immunoprecipitation with an antibody to one heavy chain, HCβ [Fowkes and Mitchell, 1998]. More recently, however, analysis of the assembly state of ODAs in the cytoplasm of the *Chlamydomonas pf22* mutant (which is defective in a cytoplasmic assembly factor) led to the unanticipated result that our anti-HCβ monoclonal antibody was unable to interact with its target epitope in non-denatured extracts of this mutant strain, even though HCβ is present in the cytoplasm at normal abundance [Mitchison et al., 2012]. In that study, we used an IC2 subunit modified to contain a single internal HA tag as an alternative target for IP purification of ODA complexes. To improve IP purifications and further assure that the IP tag has no affect on dynein assembly or function, we have now created another tagged IC2 gene construct, driven by a cloned copy of the endogenous IC2 promoter, in which three MYC epitopes were appended to the 3' end of the IC2 coding region. Cells that express this gene in an IC2 null background (*oda6*) showed wild type levels of IC2 assembly (Fig. 7D), and normal flagellar motility, consistent with a recent report of a similarly tagged IC2 gene [Oda et al., 2013]. We then introduced the *oda8* mutation into this IC2-MYC tagged strain and used an anti-MYC monoclonal antibody to IP the IC2-MYC protein from cytoplasmic extracts. IP of IC2 from *oda8* cytoplasm resulted in co-precipitation of IC1 and all three ODA heavy chains (Fig. 7E). The amount of IC1 co-precipitated was identical in wild type and *oda8* samples, however, the amount of each heavy chain co-precipitated from *oda8* cytoplasm was reduced to about half of its wild type level. Therefore, ODA subunits in *oda8* cytoplasm either have not fully assembled, or they have assembled into complexes that partially dissociate during the immunopurification wash steps. Taken together, these data show that ODA complexes in *oda8* cytoplasm have not pre-assembled to the same extent as in wild type cytoplasm, and cannot bind with normal affinity to axonemal docking sites. Therefore at least one function for the ODA8 protein involves a step in the cytoplasm that must be completed in order for assembled dynein complexes to interact productively with binding sites on axonemes, either *in vitro* or *in vivo*.

## Discussion

Here we provide evidence that the *Chlamydomonas DLU2* gene, previously identified by comparative genomic analysis as *MOT37* [Merchant et al., 2007] and as encoding a homolog of vertebrate LRRC56, is mutated in *oda8*. The distribution of this gene is not uniform among all organisms with motile cilia or flagella. Instead, LRRC56 homologs have been lost both in species that do not have ODAs and also in species that retain ODAs but that do not depend on IFT for axonemal assembly, supporting a role for this protein in IFT-based dynein transport. Further support for this view comes from analysis of amoeboid to flagellate transformation in *Naegleria*, where expression studies identified an ODA8 homolog (Friggin) as the product of a gene upregulated early during ciliogenesis, along with some axonemal and many centriole-associated proteins [Fritz-Laylin and Cande, 2010]. When expressed exogenously in cultured HeLa and U2OS cell lines, Friggin associated specifically with mother centrioles, a pattern that may reflect an IFT-related localization, since IFT proteins are also enriched around basal bodies [Cole et al., 1998] and mother centrioles [Goetz et al., 2012].

The overall fractionation of ODA8 between the flagellar and cytoplasmic compartments in *Chlamydomonas* is typical of IFT subunits, and of IFT-associated ODA assembly factor ODA16 (Fig. 3C), supporting the possible involvement of ODA8 in an IFT-dependent step in ODA assembly. However, the distribution of ODA8 during flagellar fractionation departs from that of typical IFT proteins, as about half of ODA8 remains axoneme-associated whereas most IFT subunits [Cole et al. 1998] and ODA16 [Ahmed and Mitchell, 2005] fractionate as matrix components. IFT proteins and ODA16 both have a very characteristic distribution by immunofluorescence analysis, with a high concentration of protein around basal bodies and a patchy distribution along flagella. Unfortunately the apparently much lower absolute concentration of ODA8HA has so far prevented use of this approach to determine whether ODA8 has a similar distribution. ODA16 supports ODA assembly through a specific interaction with an IFT-B protein, IFT46 [Hou et al., 2007; Ahmed et al., 2008], and has been described as an adaptor protein to aid ODA transport by IFT, although an actual direct interaction between ODA complexes and ODA16, or between ODA complexes and IFT complexes, has not yet been observed, and so the exact role of ODA16 in promoting ODA trafficking remains to be explored. ODA8 does not appear to function in precisely the same process as ODA16, however, as we [Ahmed and Mitchell, 2005] have shown that *oda16* complements in dikaryons with *oda5*, *oda8* and *oda10* mutations. In addition, the flagellar abundance of ODA16 was not altered by the loss of ODA8 (Fig. 7A), thus the ODA assembly defect in *oda8* flagella is not directly due to a disruption of the interaction between ODA16 and IFT particles. Finally, our previous result that dynein from the cytoplasm of *oda16* cells binds similarly to dynein from wild type cytoplasm onto *oda2* axonemes *in vitro* [Ahmed et al., 2008], whereas dynein from *oda8* cytoplasm does not (Fig. 7C), also shows that the role of ODA8 is different from the role of ODA16, and suggests that ODA8 functions at an earlier step than ODA16 in the pathway of dynein assembly. One other ODA assembly factor has been localized to the matrix compartment, FBB18, but unlike ODA8 or ODA16, FBB18 accumulates to higher than wild type levels in mutants with impaired motility, including various *oda* strains [Austin-Tse et al., 2013]. It thus appears that late stages of ODA assembly require several factors that may work independently of each other, and that define previously uncharacterized steps in ODA assembly.

Localization of ODA8 to flagella (Fig. 3C) and the dependence of this flagellar localization on the *ODA5* and *ODA10* gene products (Fig. 4A) are both consistent with a function for ODA8 in dynein assembly that could depend upon a physical interaction with ODA5 and ODA10 [Kamiya, 1988; Fowkes and Mitchell, 1998; Wirschell et al., 2004; Dean and Mitchell, 2013]. However, distribution of ODA8 nearly equally between axonemal and matrix fractions (Fig. 5A) departs from those expectations, as neither ODA5 [Wirshcell et al., 2004] nor ODA10 [Dean and Mitchell, 2013] appear in matrix fractions. In addition, ODA10 (unlike ODA8) shows nearly equal abundance in flagella and cytoplasm [Dean and Mitchell, 2013], and a very low level of ODA8 is still present in flagella of *oda5* and *oda10* mutant strains (Fig. 4A). Since ODA8 is present in near-normal levels in flagella of outer arm dynein subunit mutant *oda6* (Fig. 4B), the near-absence of ODA8 from *oda5* and *oda10* flagella is not due to absence of outer arm dynein in flagella but due more specifically to the *oda5* and *oda10* mutations. These findings can be reconciled if interaction between ODA8 and an ODA5/10 complex occurs primarily in the cytoplasm rather than in the flagellum.

Binding assays (Fig. 6A) indicate that strong attachment of ODAs to axonemes under *in vitro* conditions does not need ODA8, even though ODA8 is essential for normal assembly of ODAs onto axonemes *in vivo*. We [Dean and Mitchell, 2013] have shown that ODA10, although extracted from axonemes by half molar salt along with dynein, separates from dynein during sucrose gradient sedimentation and is also not needed for *in vitro* binding of outer arm dynein to axonemes. One possible explanation of these binding results would be that dynein is modified during *in vivo* assembly into a form that no longer requires ODA8 or ODA10 for axonemal re-binding. Such a modification could conceptually be done in the cytoplasm, during transport to the flagellar assembly sites, or at the time of final assembly onto doublet microtubules. Based on the greatly reduced ability of dynein present in *oda8* cytoplasmic extracts to bind onto axonemes, and on the reduced level of heavy chains seen to co-IP with intermediate chains from cytoplasmic extracts of *oda8* cells (Fig. 7C), we conclude that the initial role of ODA8 occurs during maturation of dynein in the cytoplasm and before its transport into the flagellar compartment. As discussed above, the presence of some ODA8 in flagellar matrix fractions, the similarity of its distribution between flagella and CB to that of IFT proteins, and the phylogenomic distribution of ODA8 homologs, all argue that this protein may also function during IFT-dependent ODA assembly steps, which could include attachment of ODAs to IFT complexes at the flagellar base as well as co-transport with ODAs during their IFT-dependent entry into flagella.

**Figure 8:**
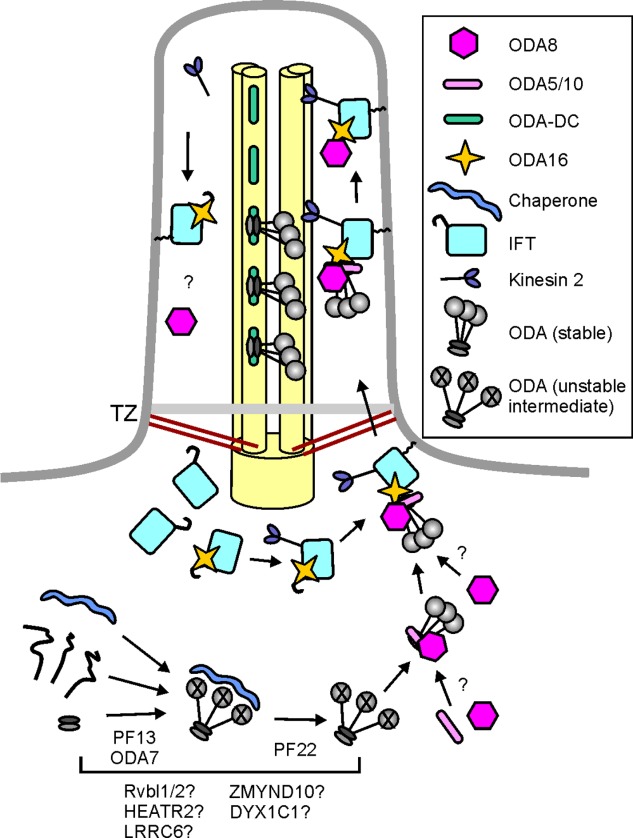
Diagram summarizing hypothesized steps in ODA assembly and transport, and a proposed role for ODA8. Initial synthesis of ODA subunits and dimerization of intermediate chains is followed by folding and co-assembly of heavy chains with intermediate chains, dependent on the action of ODA7 [Duquesnoy et al., 2009] and PF13 [Omran et al., 2008]. Several other cytoplasmic dynein assembly factors may function at this step. Exposure of a heavy chain epitope in a PF22-dependent step [Mitchison et al., 2012] is modeled as chaperone removal. ODA8 transforms an assembled, but unstable intermediate into a stable cytoplasmic storage form, which is then able to interact with IFT particles, aided by IFT-associated assembly factor ODA16. An ODA5/10 complex is hypothesized to interact with ODAs in the cytoplasm and aid ODA8 function. Once ODAs are transported to flagella, they are released from IFT particles, dissociate from ODA5/10 and ODA8, and bind through interaction of the IC dimer with ODA-DC complexes that have assembled independently on A-tubules of outer doublets.

Our working hypothesis is that ODA8 functions as an assembly factor in the cytoplasm and enables dynein to attain a form that is competent for transport and binding, and in turn ODA5 and ODA10 are needed for ODA8 function, and aid ODA8 transport to the flagellar compartment. A diagram that summarizes these relationships, and further speculates on specific functions of other dynein assembly factors, is presented in Fig. 8. Here we hypothesize that the ODA5/10 complex, which likely resembles the ODA-DC, could act as a scaffold to aid interaction of ODA8 with an unstable ODA intermediate and generate a stable ODA complex in the cytoplasm. This step could occur earlier in the assembly process than shown in the diagram and still be consistent with available data. The presence of ODA8 is hypothesized to be essential for interaction of ODA complexes with IFT particles, which in turn depends on the exposed N-terminal domain of IFT46 [Hou et al., 2007; Taschner et al., 2014], represented by a hook on the IFT complex in Fig. 8. IFT transport is enhanced by IFT46-interacting adaptor ODA16 [Ahmed et al., 2008], which depends on IFT but not on ODA8 for flagellar localization. Other cytoplasmic factors that may function during early steps include Rvbl1/2 (Pontin/Reptin) [Zhao et al., 2013], HEATR2 [Horani et al., 2012], LRRC6 [Kott et al., 2012; Horani et al., 2013; Moore et al., 2013; Zariwala et al., 2013; Zhao et al., 2013], ZMYND10 [Moore et al., 2013; Zariwala et al., 2013] and DYX1C1 [Tarkar et al., 2013], but only HEATR2 function has been assessed in *Chlamydomonas* and the stage at which it functions has not been determined. Difficult to reconcile with this model is the observed independent transport of ODA5, ODA8 and ODA10 into flagella in the absence of ODAs, and of ODA5 and ODA10 in the absence of ODA8. Additional work will be needed to determine whether ODA8 physically interacts with ODA5 or ODA10, either in the cytoplasm or the flagellum, and whether ODA8 plays a direct role in IFT-mediated ODA transport.

ODA8/LRRC56 represents an addition to a rapidly expanding list of factors needed for ODA assembly. Does the ODA represent a unique axonemal component that requires more assembly factors than other IFT-dependent axonemal cargos? Cytoplasmic assembly factors have been characterized that show specificity for select isoforms of *Chlamydomonas* inner dynein arms [Yamamoto et al., 2010] or for some inner dynein arm isoforms as well as ODAs [Omran et al., 2008; Mitchison et al., 2012], showing that this step is not ODA-specific. Relatively less is known about specific interactions of axonemal assembly cargos with IFT particles [Bhogaraju et al., 2013], but the analysis of *Chlamydomonas* assembly defective mutants suggest that the IFT-associated DYF13 protein may aid transport of several inner dynein arms, and possibly other motility-related proteins [Ishikawa et al., 2014] and additional loci are important for trafficking of I1 inner dynein arms [Viswanadha et al., 2014], and radial spokes [Alford et al., 2013], and the LC8 protein has been specifically implicated in late stages of radial spoke assembly and binding onto axonemal docking sites [Diener et al., 2011; Gupta et al., 2012]. Continued analysis of these assembly pathways should ultimately identify common themes in IFT-dependent flagellar assembly.

## Materials and Methods

### Strains and Culture

*Chlamydomonas reinhardtii* wild-type strains 137c, RFLP strain S1D2, and mutant strains *arg7*, *oda5*, *oda6*, *oda8*, and *oda10* were obtained from the Chlamydomonas Genetics Center (Univ. Minn., St. Paul, MN). Mutant strains *pf28* (an allele of *oda2*) and *oda16* were generated in previous studies and are available from the Genetics Center. Double mutant strains were created by standard crosses and tetrad analysis. Additional crosses were used to introduce epitope tagged genes (ODA8HA, IC2MYC) into these mutant backgrounds. All strains were maintained on agar solidified minimal M medium supplemented as needed with 0.05% l-arginine. For high-density liquid cultures and for transformation experiments, cells were grown on acetate-enriched minimal medium.

### Cloning and Epitope Tagging

To initiate a walk to the *ODA8* locus, strain *oda8arg7* was crossed with wild type strain S1D2, and random *oda8* progeny were selected on minimal medium. All selected strains contain a crossover between the *ARG7* and *ODA8* loci, which are separated by about 15 cM on chromosome 1. DNA prepared from these strains was used for genomic Southern blots or for amplification with marker-specific primers to map the relative positions of molecular markers and *oda8*. All markers used (CNA73, GBP1 and PBT302) have been previously described [Kathir et al., 2003].

Genomic sequences surrounding the CNA73 marker, as reported in the JGI *Chlamydomonas* genome sequence version 2.0 (http://genome.jgi-psf.org/chlre2/chlre2.home.html), were examined to identify candidate *ODA8* genes, and a BAC clone spanning the MOT37 gene was used to transform an *oda8arg7* strain. After determining that 17.5 kb BAC clone PTQ12187 (= 32F23 in plate/well nomenclature) could rescue the *oda8* mutation, smaller genomic clones were isolated by hybridization screening of a phage genomic library in EMBL4 with a BAC fragment. An 8.9 kb *Bam HI-Bam HI* fragment and a 6.8 kb *XmaI-BamHI* fragment that spanned the predicted gene were subcloned from a phage into pBlusescript II (Stratagene) to create pODA8B-B and pODA8X-B. The intron/exon structure of this gene was confirmed by RT-PCR analysis (primer sequences available on request) and by examination of RNA-Seq data (http://genomes.mcdb.ucla.edu/Cre454/).

To add seqeunces encoding three copies of the HA epitope tag to the C-terminus of the *ODA8* coding region, the 3'portion of the gene was cloned as a 2.9 kb *Sph*I-*Hin*dIII fragment into vector pSP72 and a *Stu*I restriction site was introduced near the end of the coding sequence using a QuickChange Mutagenesis kit (Stratagene). The 3' UTR was remove by *Stu*I-*Hin*dIII digestion and replaced by a 735 bp *Stu*I-*Hin*dIII fragment from plasmid pGenLinkerHA, spanning three HA epitopes and the 3' end of the *PsaD* gene [Duquesnoy et al., 2009]. The 5' end of the *ODA8* gene was then introduced between *Eco* RI and *Sph*I sites to create plasmid pODA8HA.

Constructs encoding IC2 tagged with one copy of the HA epitope, which are expressed from the native IC2 promoter, have been previously described [Mitchison et al., 2012]. To create an IC2 with three copies of the Myc epitope at the C-terminus, the IC2-HA construct was opened at a unique *BamHI* site near the stop codon, and a fragment encoding three copies of a Myc epitope in *Chlamydomonas* codon bias, synthesized by Integrated DNA Technologies (Coralville, IA), was inserted using a Gibson overlap cloning kit (New England Biolabs, Ipswich, MA). A 1038 bp *NdeI – NheI* restriction fragement spanning the HA epitope was removed and replaced with the identical length fragment from an untagged copy of the gene. As expressed, the last 15 residues of IC2 are replaced by a 9 amino acid linker followed by the three Myc epitopes.

Flagellar beat frequency of strains expressing transgenes was measured by tuning the flash rate of a stroboscopic illumination source while imaging cells under darkfield with a Zeiss Axioskop (Carl Zeiss, Jena, Germany). Swimming speed was determined from video files collected at 30 fps with a 10x objective under darkfield illumination, using CellTrak 1.5 software (Motion Analysis, Santa Rosa, CA).

### Sequence Comparisons

To identify ODA8 orthologs, *Chlamydomonas* protein sequences were used for BLASTp searches of representative eukaryotic genomes at NCBI including *Dictyostelim discoideum, Batrachochytrium dendrobatitis, Homo sapiens, Ciona intestinalis, Drosophila melanogaster, Caenorhabditis elegans, Selaginella moellendorffi, Physcomitrella patens, Ostreococcus lucimarinus, Plasmodium flaciparum, Eimeria tenella, Thalassiosira pseudonanna, Phytophthora infestans*, and *Naegleria gruberi*. The highest hits were used for a reciprocal BLASTp screen of the *Chlamydomonas reinhardtii* genome to identify orthologs. Weak hits with the *Chlamydomonas* sequence were also tested against the *Homo sapiens* genome. When no significant hit was found in a selected genome, the initial BLASTp search was repeated with the genomes of closely related genera (when available) to account for potential errors in genomic sequence assemblies. All results were categorized as significant (showing the presence of an ortholog) only if a single high-scoring sequence was observed and it generated a reciprocal best hit in the *Chlamydomonas* genome. The E-value scores are reported in Supporting Information Table I. Sequences with relatively weak *E*-value scores were still selected as orthologs if they generated a reciprocal best hit in *Chlamydomonas*, but their greater sequence divergence was noted through color coding of results in Fig. 2 and Supporting Information Table I.

### Flagellar Isolation and Fractionation, and ODA Binding Assays

Cells grown in liquid meduim were concentrated on a Pellicon filtration apparatus (Millipore Corp, Bedford, MA) and deflagellated by treatment with dibucaine as previously described [Witman 1986]. All subsequent steps were at 4°C or on ice. Flagella were purified by differential centrifugation and rinsed once in HMDEK (30 mM Hepes, 5 mM MgSO_4_, 1 mM DTT, 0.5 mM EGTA, 25 mM potassium acetate, 1 mM phenylmethylsulfonyl fluoride, pH 7.4) before preparation for SDS-PAGE (or further fractionation). To remove membranes, flagella were resuspended in HMDEK and then mixed with an equal volume of HMDEK containing a detergent, either nonidet-P40 (Fluka) or octylglucopyranoside (Sigma) as indicated in the “Results” section. After trituration with a micropipet, samples were pelleted in a microfuge at top speed for 10 min. For gel analysis pellets were resuspended to the same volume as supernatant solutions and mixed with an equal volume of 2× SDS sample buffer. For dynein extraction, demembranated axonemes were resuspended in HMDEK supplemented with the indicated concentrations of salts. All extractions proceeded for 30 min on ice, followed by centrifugation for 10 min at top speed (16,000*g*) in a microfuge. Salt extracts were dialyzed vs. HMDEK and clarified (16,000*g*, 20 min) before further use in binding assays.

To compare the abundance of proteins in CB and flagella, cells were deflagellated by pH shock [Lefebvre, 1995]. Whole cell (WC) and cell body samples for blotting were precipitated with acetone and dissolved in 1× SDS sample buffer. Soluble cytoplasmic extracts for blotting and immunoprecipitation were prepared by glass bead homogenization of protoplasts, as previously described [Ahmed and Mitchell, 2005].

Dynein binding assays were performed in HMDEK by mixing a fixed volume of axonemes with a fixed volume of an ODA containing extract. All extracts for a given experiment were adjusted to the same concentration of the ODA IC2 subunit, based on preliminary blot analysis. Because the two intermediate chains (IC1 and IC2) of *Chlamydomonas* ODA complexes interact as a heterodimer [Mitchell and Rosenbaum, 1986], form the main interaction site of ODA complexes with axonemal binding sites [King et al., 1991; Owa et al., 2014], but fail to bind tightly to axonemes unless tightly complexed with both beta and gamma heavy chains [Takada and Kamiya, 1994], the amount of IC2 bound was used as a measure of ODA binding. After incubation for 1 h on ice, axonemes were pelleted and prepared directly for SDS-PAGE, or washed in HMDEK before sample preparation, as noted in the Results section. Blots of bound proteins were probed for IC2.

### Immunoprecipitation and Blotting

Dynein heavy chains (average *M*_r_ = 500,000) and Benchmark Protein Ladder (Life Technologies) were used as protein size standards. For immunoblotting, proteins were transferred to Immobilon-P membranes (Millipore Corp). Rabbit anti-NAB1 was purchased from Agrisera (Vannas, Sweden). Affinity-purified rabbit anti-HCα [Fowkes and Mitchell, 1998] and anti-ODA16 [Ahmed and Mitchell, 2005], and mouse monoclonal anti-HCβ, anti-IC1 and anti-IC2 [Mitchell and Rosenbaum, 1986] have been previously described. Monoclonal anti-HCγ [King et al., 1985] was provided by Steven King (Univ. Conn), rabbit polyconal anti-IFT46 [Hou et al., 2007] by Joel Rosenbaum (Yale Univ.) and rabbit polyclonal anti-IC140 [Yang and Sale, 1998] by Winfield Sale (Emory Univ.). HA epitopes were detected with rat monoclonal 3F10 (Roche), Myc epitopes with mouse monoclonal 9E 10 (Developmental Studies Hybridoma Bank, Univ. Iowa) and acetylated tubulin with mouse monoclonal 6-11B-1 (Sigma, St. Louis, MO). Antibodies were detected with peroxidase-labeled secondary antibodies (Sigma) using Clarity Western ECL reagents (BioRad, Hercules, CA) and detected either by radiography on film, or with a digital camera on a Chemidoc MP Imager (Bio-Rad). Relative band intensity was determined using Chemidoc software. For immunoprecipitations, extracts were diluted with HMDEK50 buffer (HMDEK with the concentration of KAc increased to 50 mM) to provide equal concentrations of the target antigen, and pre-cleared with un-derivitized agarose beads. IC2-MYC was precipitated by incubation of extracts for 1 h at 4°C with Anti-Myc 9E 10 pre-coupled to agarose beads (Sigma), washed, and released from beads by boiling in SDS sample buffer. Blotted proteins were detected with True Blot secondary antibodies (Rockland Immunochemicals, Limerick, PA).

## Abbreviations

CB: cell bodies
DC: docking complex
FL: flagella
IFT: intraflagellar transport
LRR: leucine-rich repeat
ODA: outer dynein arm
ODA-DC: outer arm dynein docking complex
OG: octylglucpyranoside
PCD: primary ciliary dyskinesia
WC: whole cell

## References

[b1] Ahmed NT, Gao C, Lucker BF, Cole DG, Mitchell DR (2008). ODA16 aids axonemal outer row dynein assembly through an interaction with the intraflagellar transport machinery. J Cell Biol.

[b2] Ahmed NT, Mitchell DR (2005). ODA16p, a *Chlamydomonas* flagellar protein needed for dynein assembly. Mol Biol Cell.

[b3] Alford LM, Mattheyses AL, Hunter EL, Lin H, Dutcher SK, Sale WS (2013). The *Chlamydomonas* mutant *pf27* reveals novel features of ciliary radial spoke assembly. Cytoskeleton (Hoboken).

[b4] Austin-Tse C (2013). Zebrafish ciliopathy screen plus human mutational analysis identifies C21orf59 and CCDC65 defects as causing primary ciliary dyskinesia. Am J Hum Genet.

[b5] Baron DM, Kabututu ZP, Hill KL (2007). Stuck in reverse: loss of LC1 in *Trypanosoma brucei* disrupts outer dynein arms and leads to reverse flagellar beat and backward movement. J Cell Sci.

[b6] Benashski SE, Patel-King RS, King SM (1999). Light chain 1 from the *Chlamydomonas* outer dynein arm is a leucine-rich repeat protein associated with the motor domain of the gamma heavy chain. Biochemistry.

[b7] Bhogaraju S, Engel BD, Lorentzen E (2013). Intraflagellar transport complex structure and cargo interactions. Cilia.

[b8] Briggs LJ, Davidge JA, Wickstead B, Ginger ML, Gull K (2004). More than one way to build a flagellum: comparative genomics of parasitic protozoa. Curr Biol.

[b9] Casey DM, Inaba K, Pazour GJ, Takada S, Wakabayashi K, Wilkerson CG, Kamiya R, Witman GB (2003). DC3, the 21-kDa subunit of the outer dynein arm-docking complex (ODA-DC), is a novel EF-hand protein important for assembly of both the outer arm and the ODA-DC. Mol Biol Cell.

[b10] Cole DG, Diener DR, Himelblau AL, Beech PL, Fuster JC, Rosenbaum JL (1998). *Chlamydomonas* kinesin-II-dependent intraflagellar transport (IFT): IFT particles contain proteins required for ciliary assembly in *Caenorhabditis elegans* sensory neurons. J Cell Biol.

[b11] Dean AB, Mitchell DR (2013). *Chlamydomonas* ODA10 is a conserved axonemal protein that plays a unique role in outer dynein arm assembly. Mol Biol Cell.

[b12] Diener DR, Yang P, Geimer S, Cole DG, Sale WS, Rosenbaum JL (2011). Sequential assembly of flagellar radial spokes. Cytoskeleton (Hoboken.).

[b13] Duquesnoy P (2009). Loss-of-function mutations in the human ortholog of *Chlamydomonas reinhardtii* ODA7 disrupt dynein arm assembly and cause primary ciliary dyskinesia. Am J Hum Genet.

[b14] Dutcher SK (2014). The awesome power of dikaryons for studying flagella and basal bodies in *Chlamydomonas reinhardtii*. Cytoskeleton (Hoboken.).

[b15] Fowkes ME, Mitchell DR (1998). The role of preassembled cytoplasmic complexes in assembly of flagellar dynein subunits. Mol Biol Cell.

[b16] Freshour J, Yokoyama R, Mitchell DR (2007). *Chlamydomonas* flagellar outer row dynein assembly protein ODA7 interacts with both outer row and I1 inner row dyneins. J Biol Chem.

[b17] Fritz-Laylin LK, Cande WZ (2010). Ancestral centriole and flagella proteins identified by analysis of *Naegleria* differentiation. J Cell Sci.

[b18] Goetz SC, Liem KF, Anderson KV (2012). The spinocerebellar ataxia-associated gene Tau tubulin kinase 2 controls the initiation of ciliogenesis. Cell.

[b19] Gupta A, Diener DR, Sivadas P, Rosenbaum JL, Yang P (2012). The versatile molecular complex component LC8 promotes several distinct steps of flagellar assembly. J Cell Biol.

[b20] Hjeij R (2013). ARMC4 mutations cause primary ciliary dyskinesia with randomization of left/right body asymmetry. Am J Hum Genet.

[b21] Hjeij R (2014). CCDC151 mutations cause primary ciliary dyskinesia by disruption of the outer dynein arm docking complex formation. Am J Hum Genet.

[b22] Hom EF (2011). A unified taxonomy for ciliary dyneins. Cytoskeleton.

[b23] Horani A (2013). LRRC6 mutation causes primary ciliary dyskinesia with dynein arm defects. PLoS ONE.

[b24] Horani A (2012). Whole-exome capture and sequencing identifies HEATR2 mutation as a cause of primary ciliary dyskinesia. Am J Hum Genet.

[b25] Hou Y, Qin H, Follit JA, Pazour GJ, Rosenbaum JL, Witman GB (2007). Functional analysis of an individual IFT protein: IFT46 is required for transport of outer dynein arms into flagella. J Cell Biol.

[b26] Hsiao YC, Tuz K, Ferland RJ (2012). Trafficking in and to the primary cilium. Cilia.

[b27] Ishikawa H (2014). TTC26/DYF13 is an intraflagellar transport protein required for transport of motility-related proteins into flagella. Elife.

[b28] Jerber J, Baas D, Soulavie F, Chhin B, Cortier E, Vesque C, Thomas J, Durand B (2013). The coiled-coil domain containing protein CCDC151 is required for the function of IFT-dependent motile cilia in animals. Hum Mol Genet.

[b29] Kamiya R (1988). Mutations at twelve independent loci result in absence of outer dynein arms in *Chylamydomonas reinhardtii*. J Cell Biol.

[b30] Kathir P, LaVoie M, Brazelton WJ, Hass NA, Lefebvre PA, Silflow CD (2003). Molecular map of the *Chlamydomonas reinhardtii* nuclear genome. Eukaryot Cell.

[b31] King SM, Otter T, Witman GB (1985). Characterization of monoclonal antibodies against *Chlamydomonas* flagellar dyneins by high-resolution protein blotting. Proc Natl Acad Sci USA.

[b32] King SM, Wilkerson CG, Witman GB (1991). The *M*_r_ 78,000 intermediate chain of *Chlamydomonas* outer arm dynein interacts with α-tubulin *in situ*. J Biol Chem.

[b33] Kott E (2012). Loss-of-function mutations in LRRC6, a gene essential for proper axonemal assembly of inner and outer dynein arms, cause primary ciliary dyskinesia. Am J Hum Genet.

[b34] Koutoulis A, Pazour GJ, Wilkerson CG, Inaba K, Sheng H, Takada S, Witman GB (1997). The *Chlamydomonas reinhardtii ODA3* gene encodes a protein of the outer dynein arm docking complex. J Cell Biol.

[b35] Kutomi O, Hori M, Ishida M, Tominaga T, Kamachi H, Koll F, Cohen J, Yamada N, Noguchi M (2012). Outer dynein arm light chain 1 is essential for controlling the ciliary response to cyclic AMP in *Paramecium tetraurelia*. Eukaryot Cell.

[b36] Lefebvre PA (1995). Flagellar amputation and regeneration in *Chlamydomonas*. Methods Cell Biol.

[b37] Lobo LJ, Zariwala MA, Noone PG (2014). Primary ciliary dyskinesia. QJM.

[b38] Loges NT (2009). Deletions and point mutations of LRRC50 cause primary ciliary dyskinesia due to dynein arm defects. Am J Hum Genet.

[b39] Loges NT (2008). DNAI2 mutations cause primary ciliary dyskinesia with defects in the outer dynein arm. Am J Hum Genet.

[b40] Merchant SS (2007). The *Chlamydomonas* genome reveals the evolution of key animal and plant functions. Science.

[b41] Mitchell DR, Rosenbaum JL (1986). Protein-protein interactions in the 18S ATPase of *Chlamydomonas* outer dynein arms. Cell Motil Cytoskeleton.

[b42] Mitchison HM (2012). Mutations in axonemal dynein assembly factor DNAAF3 cause primary ciliary dyskinesia. Nat Genet.

[b43] Moore DJ (2013). Mutations in ZMYND10, a gene essential for proper axonemal assembly of inner and outer dynein arms in humans and flies, cause primary ciliary dyskinesia. Am J Hum Genet.

[b44] Oda T, Yagi T, Yanagisawa H, Kikkawa M (2013). Identification of the outer-inner dynein linker as a hub controller for axonemal dynein activities. Curr Biol.

[b45] Olbrich H (2002). Mutations in *DNAH5* cause primary ciliary dyskinesia and randomization of left-right and asymmetry. Nat Genet.

[b46] Omran H (2008). Ktu/PF13 is required for cytoplasmic pre-assembly of axonemal dyneins. Nature.

[b47] Onoufriadis A (2014). Combined exome and whole-genome sequencing identifies mutations in ARMC4 as a cause of primary ciliary dyskinesia with defects in the outer dynein arm. J Med Genet.

[b48] Owa M, Furuta A, Usukura J, Arisaka F, King SM, Witman GB, Kamiya R, Wakabayashi K (2014). Cooperative binding of the outer arm-docking complex underlies the regular arrangement of outer arm dynein in the axoneme. Proc Natl Acad Sci U S A.

[b49] Panizzi JR (2012). CCDC103 mutations cause primary ciliary dyskinesia by disrupting assembly of ciliary dynein arms. Nat Genet.

[b50] Pennarun G, Escudier E, Chapelin C, Bridoux AM, Cacheux V, Roger G, Clément A, Goossens M, Amselem S, Duriez B (1999). Loss-of-function mutations in a human gene related to *Chlamydomonas reinhardtii* dynein IC78 result in primary ciliary dyskinesia. Am J Hum Genet.

[b51] Takada S, Kamiya R (1994). Functional reconstitution of *Chlamydomonas* outer dynein arms from alpha-beta and gamma subunits: requirement of a third factor. J Cell Biol.

[b52] Takada S, Wilkerson CG, Wakabayashi K, Kamiya R, Witman GB (2002). The outer dynein arm-docking complex: Composition and characterization of a subunit (Oda1) necessary for outer arm assembly. Mol Biol Cell.

[b53] Tarkar A (2013). DYX1C1 is required for axonemal dynein assembly and ciliary motility. Nat Genet.

[b54] Taschner M, Kotsis F, Braeuer P, Kuehn EW, Lorentzen E (2014). Crystal structures of IFT70/52 and IFT52/46 provide insight into intraflagellar transport B core complex assembly. J Cell Biol.

[b55] Viswanadha R, Hunter EL, Yamamoto R, Wirschell M, Alford LM, Dutcher SK, Sale WS (2014). The ciliary inner dynein arm, I1 dynein, is assembled in the cytoplasm and transported by IFT before axonemal docking. Cytoskeleton (Hoboken.).

[b56] Wakabayashi K, Takada S, Witman GB, Kamiya R (2001). Transport and arrangement of the outer-dynein-arm docking complex in the flagella of *Chlamydomonas* mutants that lack outer dynein arms. Cell Motil Cytoskeleton.

[b57] Wirschell M, Pazour G, Yoda A, Hirono M, Kamiya R, Witman GB (2004). ODA5, a novel axonemal protein required for assembly of the outer dynein arm and an associated adenylate kinase. Mol Biol Cell.

[b58] Witman GB (1986). Isolation of *Chlamydomonas* flagella and flagellar axonemes. Methods Enzymol.

[b59] Yamamoto R, Hirono M, Kamiya R (2010). Discrete PIH proteins function in the cytoplasmic preassembly of different subsets of axonemal dyneins. J Cell Biol.

[b60] Yang PF, Sale WS (1998). The M_r_ 140,000 intermediate chain of *Chlamydomonas* flagellar inner arm dynein is a WD-repeat protein implicated in dynein arm anchoring. Mol Biol Cell.

[b61] Zariwala MA (2013). ZMYND10 is mutated in primary ciliary dyskinesia and interacts with LRRC6. Am J Hum Genet.

[b62] Zhao L, Yuan S, Cao Y, Kallakuri S, Li Y, Kishimoto N, DiBella L, Sun Z (2013). Reptin/Ruvbl2 is a Lrrc6/Seahorse interactor essential for cilia motility. Proc Natl Acad Sci U S A.

