# Supplementary material for: Chlamydomonas Axonemal Dynein Assembly Locus ODA8 Encodes a Conserved Flagellar Protein Needed for Cytoplasmic Maturation of Outer Dynein Arm Complexes
Source: Cytoskeleton (Hoboken). 2015 Feb 7;72(1):16–28. doi: 10.1002/cm.21206 (PMC4361367; doi:10.1002/cm.21206)
Supplement: Supplementary file 1 [file cm0072-0016-sd1.pdf]

Supplemental Table 1

BLASTp E-values vs. *Chlamydomonas* proteins

**Complex**	ODA	ODA	IDA	IDA	IFTA	IFTA	IFTB	IFTB	IFTB	IFTB	ODA-DC		
**C.r. Protein**	HCγ	IC2	HCα	IC140	122	140	46	52	88	172	DC2	ODA16	ODA8
**Accession No.**	AAA50455	CAA39053	CAB56598	EDP01123	AFC88886	EDP01047	EDP01047	EDP04111	AAG37228	EDP04848	EDO97433	AAZ77789	Cre01.g043650.t1
**Genus**	**BLASTp E-values for top-scoring protein sequences**												
Dictyostelium	1E-04	2E-34	7E-10	7E-10	5E-13	9E-05	9E-03	4E-01	2E-06	2E-05	6E-01	2E-53	4E-06
Batrachochytrium	0E+00	0E+00	0E+00	4E-125	0E+00	6E-159	2E-58	2E-141	0E+00	0E+00	2E-62	3E-167	5E-27
Homo	1E-155	0E+00	0E+00	2E-110	0E+00	0E+00	5E-75	5E-139	0E+00	0E+00	1E-31	0E+00	3E-46
Ciona	3E-166	0E+00	0E+00	3E-103	0E+00	0E+00	2E-78	8E-133	0E+00	0E+00	3E-99	0E+00	2E-36
Drosophila	1E-70	1E-130	2E-42	2E-32	0E+00	2E-163	8E-22	1E-70	4E-81	0E+00	8E-23	3E-116	1E-26
Caenorhabditis	4E-03	1E-16	1E-08	2E-06	0E+00	7E-149	8E-54	7E-84	3E-144	0E+00	9E-02	3E-25	6E-09
Selaginella	5E-62	2E-25	0E+00	5E-50	0E+00	3E-25	1E-66	4E-125	0E+00	0E+00	2E-04	1E-34	5E-07
Physcomitrella	1E-62	1E-26	0E+00	8E-96	0E+00	0E+00	1E-28	1E-71	0E+00	0E+00	5E-10	3E-52	1E-06
Chlamydomonas	0E+00	0E+00	0E+00	0E+00	0E+00	0E+00	0E+00	0E+00	0E+00	0E+00	0E+00	0E+00	0E+00
Ostreococcus	1E-35	2E-19	2E-152	2E-05	8E-09	1E-04	4E+00	7E-01	1E-07	7E-04	1E-01	1E-25	5E-05
Plasmodium	5E-21	3E-83	2E-26	4E-17	2E-11	5E-01	5E+00	2E+00	2E-04	1E-01	9E+00	3E-26	4E-09
Eimeria	5E-51	9E-133	1E-45	3E-17	3E-09	1E-01	1E+00	3E-01	1E+00	2E-03	3E-13	6E-23	2E-05
Thallassiosira	0E+00	1E-168	2E-82	2E-31	5E-05	8E-02	1E-23	1E-96	8E-133	7E-04	5E-69	2E-30	2E-06
Phytophthora	0E+00	2E-150	0E+00	2E-72	0E+00	0E+00	3E-75	4E-131	0E+00	0E+00	1E-88	0E+00	3E-33
Naegleria	0E+00	4E-128	1E-161	3E-71	0E+00	0E+00	5E-58	2E-132	4E-180	0E+00	2E-86	0E+00	6E-39

highly significant similarity (best reciprocal BLAST hit)

weakly significant similarity (best reciprocal BLAST hit)

no significant similarity (NOT best reciprocal BLAST hit)
